# Knowledge Mapping of *Angelica sinensis (Oliv.) Diels* (Danggui) Research: A Scientometric Study

**DOI:** 10.3389/fphar.2020.00294

**Published:** 2020-03-13

**Authors:** Cuncun Lu, Ming Liu, Wenru Shang, Yuan Yuan, Meixuan Li, Xiuxiu Deng, Huijuan Li, Kehu Yang

**Affiliations:** ^1^Evidence-Based Medicine Center, School of Basic Medical Sciences, Lanzhou University, Lanzhou, China; ^2^Evidence-Based Social Science Center, School of Public Health, Lanzhou University, Lanzhou, China; ^3^School of Public Health, Fudan University, Shanghai, China; ^4^Clinical College of Chinese Medicine, Gansu University of Traditional Chinese Medicine, Lanzhou, China; ^5^Central Laboratory, Hospital of Chengdu University of Traditional Chinese Medicine, Chengdu, China

**Keywords:** traditional Chinese medicine, *Angelica sinensis (Oliv.) Diels*, scientometric, HistCite, CiteSpace

## Abstract

**Background:**

Traditional Chinese medicine (TCM) has been widely accepted and applied worldwide, and many publications related to *Angelica sinensis (Oliv.) Diels* (AS, Chinese name is “*Danggui”*) have been published. However, to date, there has not been a scientometric study to systematically analyze the intellectual landscape and emerging research trends regarding AS. Therefore, we performed a scientometric study to address this gap.

**Methods:**

Publications related to AS published from 2009 to 2018 were identified and selected from the Web of Science (WoS) Core Collection on May 30, 2019 using relevant keywords. HistCite, CiteSpace, and Excel 2016 software tools were used to conduct this scientometric study.

**Results:**

Seven hundred and sixty-seven articles (including 717 primary articles and 60 review articles) and their cited references were included and analyzed. The majority of publications (*N* = 565, 73.7%) were published in mainland China, with Nanjing University of Chinese Medicine contributing the most publications (*N* = 42, 5.5%). The first core journal was *Journal of Ethnopharmacology* (*N* = 58, 7.6%; impact factor = 3.414). The identification and assessment of active components (like ferulic acid) of AS and their pharmacological actions (such as immunomodulatory effects) are the current research foci for AS research.

**Conclusion:**

The present scientometric study provides an overview of the development of AS research over the previous decade using quantitative and qualitative methods, and this overview can provide references for researchers focusing on AS.

## Introduction

With the introduction of evidence-based medicine (EBM) and the development of traditional Chinese medicine (TCM) research, TCM has gradually gained acceptance throughout the world (Chen Y. L. et al., 2018). Herbs are one of the most commonly used interventions for the treatment of diseases in TCM (Flower et al., 2014; Zhang K. J. et al., 2019). While the *Angelica sinensis (Oliv.) Diels* (AS), which is known as Danggui in Chinese, is mainly cultivated in northwestern China and is one of the most important herbs in TCM (Zou et al., 2019). Due to its biological effects, AS has become increasingly popular as a health product in China, Japan, and Korea (Wei et al., 2016). According to TCM theory, AS has the effect of invigorating the blood, promoting circulation, lubricating the intestines, and regulating menstruation (Wei et al., 2016, 2019; Tian et al., 2017). Modern pharmacological studies have revealed that compounds identified and isolated from AS have a variety of pharmacological actions, including anti-inflammatory, anti-tumor, immunostimulatory, hormone-regulation, anti-hepatotoxic, neuroprotective, anti-aging and anti-cardiovascular diseases effects (Ooi and Liu, 2000; Chao and Lin, 2011). AS can be used alone, but is more often prescribed in combination with other medicines in a formulation, such as Siwu (SW) (Hu and Guo, 2017; Feng and Shi, 2019) and Danggui buxue (DGBX) decoration (Lin et al., 2017; Zhao et al., 2017). For example, SW consists of four medicines: (1) AS, (2) Chuanxiong [CX, *Conioselinum anthriscoides ‘Chuanxiong’ (Apiaceae)*], (3) Baishao [BS, *Paeonia lactiflora Pall. (Paeoniaceae)*], and (4) ShuDiHuang [SDH, *Rehmannia glutinosa (Gaertn.)DC.(Orobanchaceae)*], which are used to treat constipation and anemia (Hu and Guo, 2017; Feng and Shi, 2019). The other one common formulation, DGBX, consists of two herbs, AS and Huangqi [HQ, *Astragalus mongholicus Bunge (Fabaceae)*], and is used to treat anemia and menopausal symptoms, among other diseases (Lin et al., 2017; Zhao et al., 2017).

Scientometric analysis is a method that has been widely employed to analyze research frontiers and development trends of unique research fields or subjects (Xiao et al., 2017; Miao et al., 2018; Chen et al., 2019; Lu et al., 2019). For example, Xiao et al. (2017) employed this method to study organic photovoltaic technology (OPV) and found that the emerging trends of OPV research included inverted device structure and tandem solar cells. Miao et al. (2018) applied this method to analyze hepatocellular carcinoma (HCC) research and found that transarterial chemoembolization, cancer stem cells and the epithelial-mesenchymal transition are the major research foci of HCC. In a published work, we analyzed the top 100 most-cited publications related to network pharmacology (NP), and found that NP focused on cardiovascular diseases and cancers (Lu et al., 2019). Although many publications related to AS have been published in the peer-reviewed journals, no research to date has systematically analyzed the intellectual landscape and emerging trends of AS research using scientometric analysis. Therefore, the purpose of this study was to map the knowledge landscape and development trends of AS research using scientometric analysis.

## Materials and Methods

### Data Sources

We searched the Web of Science (WoS) Core Collection on May 30, 2019 to collect publications related to AS. To investigate recent development trends in AS research, the time span was set from 2009 to 2018, and publications types were limited to primary articles or review articles. The search strategy was as follows: Topic = “angelica sinensis” OR “Danggui” OR “Dang gui” OR “Chinese angelica.” All identified records were downloaded from the WoS database on May 30, 2019 and imported into scientometric software tools for further analysis.

### Statistical Analysis

HistCite (12.03.07) is a software tool created by Garfield et al. (2006) that is used for scientometric analysis and visualization of scientific literature. In the present study, we used HistCite to identify the annual output (number of articles) and top countries/regions, institutions and core journals of AS research. Journals impact factor (IF) was collected from Journal Citation Reports (2018, Thomson Reuters^1^). We did not analyze active authors in AS research due to the possibility of name homonyms. CiteSpace (5.4.R1) is a scientometric software tool that primarily focuses on co-citation analyses and is able to create a variety of map visualizations, including network visualization, cluster visualization, and dual-map overlay (Chen, 2017). In the created visualization maps, each node represents an entity with its size proportional to the number of citations and the link between two nodes representing the strength of relationship between the entities. Additionally, different colors correspond to different time periods, ranging from cold colors to warm colors (for example, from purple to orange). In CiteSpace, betweenness centrality (BC) is a critical parameter that measuring measures the scientific importance of the nodes, and nodes with high betweenness centrality (BC ≥ 0.1) usually are highlighted using purple rings. CiteSpace also analyzes the intellectual landscape by conducting co-citations analysis and identifying emerging topics in terms of articles or keywords with strong citation bursts. In this scientometric study, CiteSpace was used to construct network visualization and cluster visualization of co-citations analysis, while also creating the dual-map overlay of journals and detecting the studies with strong with strong citation bursts. The parameters of CiteSpace were as follows: link retaining factor (LRF = 2), look back years (LBY = −1), e for top N (*e* = 2), time span (2009–2018), years per slice (1), links (strength: cosine, scope: within slices), selection criteria (Top 50). Microsoft Office Excel 2016 (Microsoft Corporation, Redmond, WA, United States) was used to manage data and create the chart of annual research output.

## Results and Discussion

### Year of Publications

**FIGURE 1:**
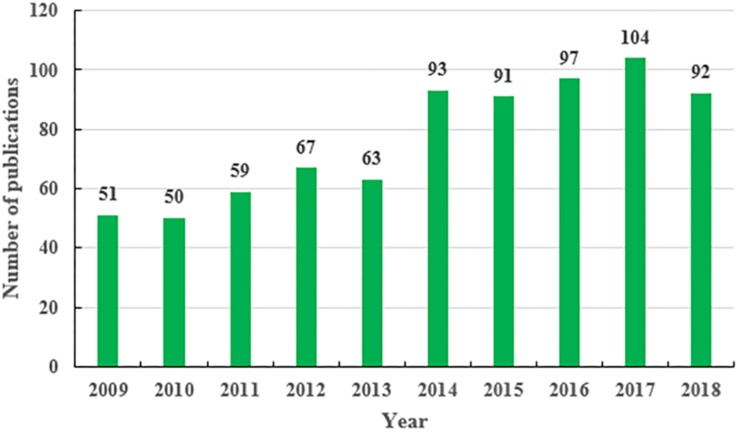
Annual output of *Angelica sinensis* research.

Seven hundred and sixty-seven studies related to AS were identified that were published from 2009 to 2018, including 717 primary articles and 60 review articles. The publication languages of identified articles were English (*N* = 759, 99.0%) and Chinese (*N* = 8, 1.0%). As is shown in Figure 1, in 2009, 2010, and 2011, the number of publications ranges from 50 to 59; in 2012 and 2013, the number of publications ranges from 63 to 67; in 2014, 2015, 2016, and 2018, the number of publications ranges from 91 to 97. The number of articles in a single year exceeded 100 in 2017 only, while the fewest studies (*N* = 50) were published in 2010. Overall, there were approximately 80 articles published each year on average, with a gradual and consistent growth trend (Figure 1). There are a number of potential reasons for the slow but consistent growth trend that we observed. For example, by only focusing on AS, we narrowed the scope of the research topic and thus limited the potential number of conducted studies (Chen et al., 2019). Additionally, the slow growth trend may be due to the complexity of TCM (Zhang W. et al., 2019), the limitations of existing experimental methods in TCM (Zhu et al., 2019), the fact that relatively fewer researchers focus on TCM, and the fact that it is difficult to achieve large breakthroughs in TCM. Despite these obstacles, the Chinese scientist *Tu YouYou* discovered and extracted artemisinin from herbal medicine [*Artemisia annua L. (Compositae-Anthemideae)*] as a treatment for malaria and won the 2015 Nobel Prize in Physiology or Medicine (Wang et al., 2018). At the same time, in recent years, a new research method of TCM, NP (or systems pharmacology) (Xie et al., 2018) and has been widely accepted and employed in recent years to investigate the mechanisms of TCM for many chronic complex conditions at the molecular, cellular, and system level, such as cardiovascular diseases, diabetes mellitus, and tumors (Li et al., 2017; Lu et al., 2019). Meanwhile, the concept of EBM has also been introduced into TCM (Luo et al., 2012; Zhao et al., 2017; Chen Y. L. et al., 2018). All of these developments are important for the modernization and internationalization of TCM, and also provide a huge potential for the development of AS and herbs research. Therefore, expanding the number of teams researching AS and other herbs in the future and increasing the corresponding capital investment are promising for improving human health.

### Countries and Institutions

The top five countries/regions involved in AS research are presented in Table 1. The most AS publications (*N* = 565, 73.7%) were published by researchers in mainland China, followed by Taiwan (China) (*N* = 89, 11.6%). Researchers in South Korea, the United States, and Japan published 51 (6.6%), 40 (5.2%), and 14 (1.8%) articles, respectively. In total, 746 institutions involved in AS research, and the top five institutions are also presented in Table 1. The top five institutions are all located in China, with Nanjing University of Chinese Medicine contributed the most AS publications (*N* = 42, 5.5%) of all institutions, Medical University (*N* = 37, 4.8%), Hong Kong University of Science and Technology (*N* = 36, 4.7%), China Pharmaceutical University (*N* = 30, 3.9%), and China Medical University Hospital (*N* = 28, 3.7%). There are a variety of obvious reasons why all five of the top institutions publishing AS research are located in China. First, China is one of the main areas where AS is actually produced (Zou et al., 2019). Second, China is the only country that implements both TCM and Western medicine at the same time in clinical practice (Chen Y. et al., 2018). Third, AS is often prescribed by Chinese doctors due to its various pharmacological effects, such as improving immune function, promoting blood circulation, and lubricating the intestines (Wei et al., 2016, 2019). Finally, there are there are more medical institutions and doctors practicing TCM in China than anywhere else. For example, and according to the “Statistical Bulletin on the Development of Healthcare in China 2018”,^2^ there are 60,738 TCM institutions and 715,000 TCM doctors in mainland China. Therefore, it is logical that due to the increased attention and application of AS within China, more research is contributed by Chinese researchers from institutions within China.

**TABLE 1:** The top five countries/regions and institutions in *Angelica sinensis* research.

**Country/Region**	**Count**	**Institution**	**Count**	**Country/Region**
Mainland China	565	Nanjing University of Chinese Medicine	42	China
Taiwan (China)	89	China Medical University	37	Taiwan (China)
South Korea	51	Hong Kong University of Science and Technology	36	China
United States	40	China Pharmaceutical University	30	China
Japan	14	China Medical University Hospital	28	China

### Journals Analysis

**FIGURE 2:**
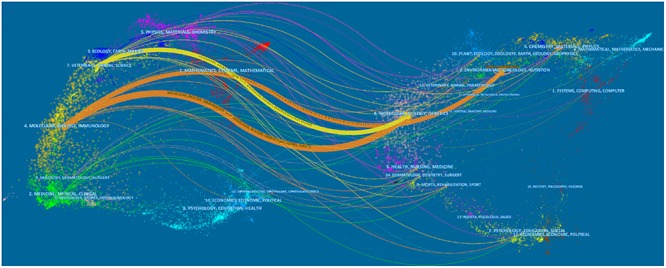
The dual-map overlay of *Angelica sinensis* research.

The five most productive journals in AS research are presented in Table 2. These five journals were published in four countries (Ireland, England, Netherlands, and Switzerland). The *Journal of Ethnopharmacology* published the greatest number of AS publications (*N* = 58, 7.6%), followed by *Evidence-Based Complementary and Alternative Medicine* (*N* = 45, 5.9%), *Carbohydrate Polymers* (*N* = 19, 2.5%), the *Journal of Pharmaceutical and Biomedical Analysis* (*N* = 19, 2.5%), and *Molecules* (*N* = 18, 2.3%). The IF of the journals publishing AS research ranged from 1.984 (*Evidence-Based Complementary and Alternative Medicine*) to 6.044 (*Carbohydrate Polymers*). As is shown in Figure 2, the dual-map overlay of journals represents the subject distribution of journals, with the left side of the graph representing citing journals, the right side of the graph indicating cited journals, and the colored lines representing the citation relationship between articles in citing journals and articles in cited journals (Chen, 2017; Miao et al., 2018). We found that there were three citation paths, including two orange paths and one yellow path. The upper yellow path, indicates that papers published in Veterinary/Animal/Science journals usually cited papers published in Molecular/Biology/Genetics journals. The middle orange path, means that papers published in Molecular/Biology/Immunology journals typically cited papers published in Environmental/Toxicology/Nutrition journals. Finally, the lower orange path, represents that papers published in Molecular/Biology/Immunology journals often cited papers published in Molecular/Biology/Genetics journals. All of above journal analyses can provide indispensable references for new researchers in the AS field who are developing their experiments and manuscripts.

**TABLE 2:** The top five journals in *Angelica sinensis* research.

**Journal name**	**Country/Area**	**Count**	**IF 2018**
Journal of Ethnopharmacology	Ireland	58	3.414
Evidence-Based Complementary and Alternative Medicine	England	45	1.984
Carbohydrate Polymers	England	19	6.044
Journal of Pharmaceutical and Biomedical Analysis	Netherlands	19	2.983
Molecules	Switzerland	18	3.06

*IF, impact factor.*

### Document Co-citation Analysis

**FIGURE 3:**
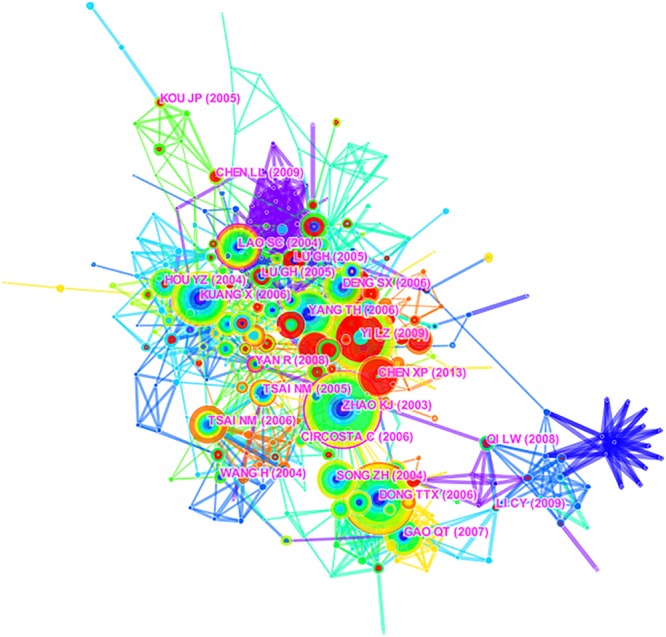
The network map of document co-citation of *Angelica sinensis* research.

**FIGURE 4:**
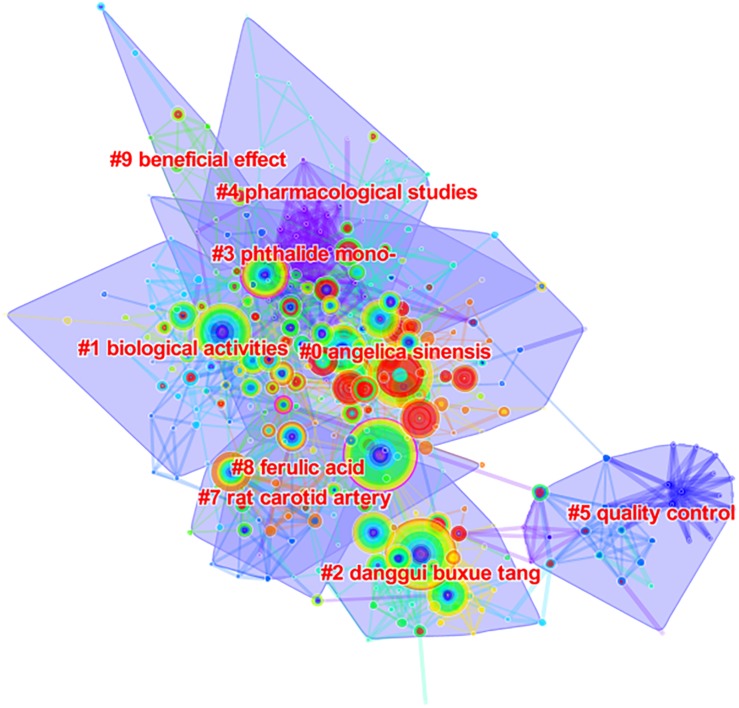
The cluster map of document co-citation of *Angelica sinensis* research.

The knowledge base and research development of AS research was characterized using the highest co-cited references and key clusters of articles, respectively (Chen, 2017; Xiao et al., 2017). A network map with 424 unique nodes and 1,510 links of document co-citation was generated (Figure 3), along with a cluster map (Figure 4, Modularity *Q* = 0.7473, Silhouette = 0.2548). For the cluster map, the nine largest clusters (small clusters were automatically filtered), included #0“Angelica sinensis,” #1“Biological activities,” #2“Danggui buxue tang,” #3“Pathalide mono-,” #4“Pharmacological studies,” #5“Quality control,” #7“Rat carotid artery,” #8“Ferulic acid,” and #9“Beneficial effect.” Purple links describe articles that were co-cited in 2009 and orange links describe articles that were co-cited in 2018. In CiteSpace, Modularity *Q* and mean Silhouette are used to measure quality of the clusters. Typically, *Q* > 0.3 and *S* > 0.5 indicate that the quality of the clusters is good (Chen, 2017; Xiao et al., 2017).

Three nodes (Zhao et al., 2003; Lao et al., 2004; Yan et al., 2008) have purple rings (Figures 3, 4), indicating they are pivot nodes and may bridge the connection between two research themes or subjects. Table 3 presents the top 10 co-cited references in AS. Among them, Zhao et al. (2003) published the paper with the highest number of citations (58 co-citations) and was published in 2003, and is also the first (BC = 0.14) of the three pivot nodes. In this paper, the authors analyzed three kinds of AS roots using molecular genetic methods and found AS roots from China contains more ferulic acid and Z-ligustilide compared to Japan and Korea. The authors concluded that different environments and storage methods led to the observed result. The paper with the second greatest number of citations (Dong et al., 2006) received 56 co-citations, was published in 2006. This study used biochemical methods to demonstrate that a specific ratio (5:1) of HQ and AS in DGBX has the largest effects, a result which coincides with typical TCM practice. The third most-cited study (Yi et al., 2009) received 52 co-citations and was published in 2009. In this study, the authors summarized the main compounds, quality control, and chemical analysis methods of AS. Kuang et al. (2006) published the fourth most-cited study, it reveieved 45 co-citations and was published in 2006. This paper confirmed that Z-ligustilide in AS has a protective effect against brain damage induced by brain ischemia. Yang et al. (2006) published the study with the fifth greatest number of citations, which received 40 co-citations and was also published in 2006. This experiment proved that the immunomodulatory function of polysaccharide contains in AS relies on the regulation of cytokines. The sixth most-cited study (Lao et al., 2004) received 38 co-citations and published in 2004. The authors found that gas chromatography–mass spectrometry combined with pressurized liquid extraction provided an effective method for analyzing AS, and also found that the level of certain compounds in three species of AS varied, a result that was similar to Zhao et al. (2003). Lao et al. (2004) is also the second (BC = 0.13) of the three pivot nodes, while the third node (BC = 0.12) of the three pivot nodes is Yan et al. (2008), where the authors reported the pharmacokinetics of ligustilide from CX in rats. Although this paper did not study AS directly, it cited several AS publications, and the “KeyWords Plus” section of this paper was labeled with “ANGELICA-SINENSIS” in the WoS database. Moreover, in TCM daily practice, AS and CX are often prescribed in one prescription at the same time, such as SW (Feng and Shi, 2019). Song et al. (2004) published the seventh greatest number of citations paper in 2004, it received 34 co-citations. The study authors identified the best conditions for extracting constituents contained in HQ and AS, and they concluded that their findings could benefit quality control of herbal medicines. The eighth most-cited study received 33 co-citations and was published in 2007 by Gao et al. (2007). This study explored the estrogenic effects of HQ and AS, and suggested that TCM needs well-defined formulations based on its characteristics. The ninth most-cited study (Tsai et al., 2006) was published in 2006, and it received 32 co-citations. This experimental study reported that the anti-tumor effects of *n*-butylidenephthalide, which is a chloroform extraction of AS. Deng et al. (2006) published the 10th most-cited paper in 2006, it received 30 co-citations. In this paper, authors reported on the isolation and identification of 21 compounds of AS and their serotonergic effects.

**TABLE 3:** The most highly cited publications in the local dataset.

**ID**	**Title**	**Year**	**Citation**	**Cluster**
Zhao et al. (2003)	Molecular genetic and chemical assessment of radix Angelica (Danggui) in China	2003	58	#2
Dong et al. (2006)	Chemical and biological assessment of a Chinese herbal decoction containing Radix Astragali and Radix Angelicae Sinensis: Determination of drug ratio in having optimized properties	2006	56	#2
Yi et al. (2009)	The analysis of Radix Angelicae Sinensis (Danggui)	2009	52	#0
Kuang et al. (2006)	Neuroprotective role of Z-ligustilide against forebrain ischemic injury in ICR mice	2006	45	#1
Yang et al. (2006)	Immunomodulatory activity of polysaccharide isolated from *Angelica sinensis*	2006	40	#0
Lao et al. (2004)	Identification and quantification of 13 components in *Angelica sinensis* (Danggui) by gas chromatography–mass spectrometry coupled with pressurized liquid extraction	2004	38	#3
Song et al. (2004)	Chemical and biological assessment of a traditional Chinese herbal decoction prepared from Radix Astragali and Radix Angelicae Sinensis: orthogonal array design to optimize the extraction of chemical constituents	2004	34	#2
Gao et al. (2007)	Danggui buxue tang - A Chinese herbal decoction activates the phosphorylations of extracellular signal-regulated kinase and estrogen receptor alpha in cultured MCF-7 cells	2007	33	#2
Tsai et al. (2006)	The natural compound *n*-butylidenephthalide derived from *Angelica sinensis* inhibits malignant brain tumor growth *in vitro* and *in vivo*	2006	32	#7
Deng et al. (2006)	Serotonergic activity-guided phytochemical investigation of the roots of *Angelica sinensis*	2006	31	#3

Among the top 10 co-cited references, four papers (Zhao et al., 2003; Song et al., 2004; Dong et al., 2006; Gao et al., 2007) belong to cluster #2, two papers (Yang et al., 2006; Yi et al., 2009) belong to cluster #0, two papers (Lao et al., 2004; Deng et al., 2006) belong to cluster #3, and the remained references (Kuang et al., 2006; Yeh et al., 2011) belong to cluster #1 and cluster #7, respectively. The clusters of AS publications are presented in Table 4. CiteSpace is able to extract cluster labels from the titles of articles that cited the cluster based on three unique parameters—latent semantic indexing (LSI), log-likelihood ratio (LLR), and mutual information (MI). Typically, LLR generates the best result by combining uniqueness and completeness of themes of a cluster (Chen, 2017; Xiao et al., 2017). Table 5 summarizes the active citing article in each cluster. In the whole network, *Q* = 0.7473, *S* = 0.2548, with the *S* is lower than the threshold value (*S* = 0.5) for cluster quality. However, within each cluster, the values of *S* were all far greater than the threshold and we therefore considered that the cluster results also to be meaningful. For these significant clusters, the largest cluster (#0“Angelica sinensis”) included 77 member papers with an average publication year of 2006. In this cluster, the most active citing paper (Liu P. J. et al., 2010) was published in 2010 and entitled “*Hematopoietic effect of water-soluble polysaccharides from Angelica sinensis on mice with acute blood loss*” and cited 11 papers of 77 members papers. This experimental study found that polysaccharides from AS can be used for mice with acute blood loss, and have potential to treat anemia, with their most-cited study being Yi et al. (2009). The second largest cluster (#1“Biological activities”) had 53 member articles with an average publication year of 2007. The most active citing paper (Li et al., 2012) in this cluster published in *Molecules* in 2012, cited 11 member papers and reviewed the research advances of CX. While the most-cited paper (Kuang et al., 2006) in the cluster did not discuss AS directly and the observed phenomenon is similar to Yan et al. (2008), where the “KeyWords Plus” field was also labeled with “ANGELICA-SINENSIS” in the WoS database, but it cited more publications related to AS. To some extent, this shows that there is a great correlation between the research on these two herbs, and this reflected the clinical practice as they are often prescribed in one prescription, simultaneously (Feng and Shi, 2019). “Danggui buxue tang” was the third largest cluster (#2), and had 43 member papers with an average publication year of 2008. The most active citing paper (Lin et al., 2017) to this cluster was published in *Journal of Ethnopharmacology* in 2017 and also cited 11 member papers. In this paper, the authors commented that herbal extract quality control and unknown mechanisms of action are the largest two problems in the TCM development, and they developed a useful strategy to reveal mechanism of TCM by using DGBX for menopausal symptoms as a case study. The most co-cited paper of this cluster was Zhao et al. (2003).

**TABLE 4:** The largest nine clusters of document co-citation in *Angelica sinensis* research.

**Cluster**	**Size**	**Silhouette**	**Year**	**Label-LSI**	**Label-LLR**	**Label-MI**
#0	77	0.693	2006	Angelica sinensis	Angelica sinensis	Angelica polysaccharide
#1	53	0.746	2007	Ligustilide	Biological activities	Cancer research mice
#2	43	0.857	2008	Danggui buxue tang	Danggui buxue tang	Ancient Chinese
#3	36	0.865	2003	Angelica sinensis	Pathalide mono-	Beta-amyloid content
#4	29	0.836	2006	Shaofu Zhuyu decoction	Pharmacological studies	Traditional Chinese Medicinal plant angelica
#5	29	0.961	2006	Of-flight mass spectrometry	Quality control	Macroporous resin chromatography
#7	24	0.886	2006	*N*-butylidenephthalide	Rat carotid artery	Myofibroblasts activation
#8	20	0.841	2007	Angelica sinensis	Ferulic acid	Altered expression
#9	23	0.893	2008	Memory	Beneficial effect	Beta-amyloid content

*LSI, latent semantic indexing; LLR, log-likelihood ratio; MI, mutual information.*

**TABLE 5:** The most active citing article in each cluster.

**ID**	**Title**	**Year**	**Coverage**	**Cluster**
Liu P. J. et al. (2010)	Hematopoietic effect of water-soluble polysaccharides from *Angelica sinensis* on mice with acute blood loss	2010	11	#0
Li et al. (2012)	Advances in the chemical analysis and biological activities of chuanxiong	2012	11	#1
Lin et al. (2017)	Danggui Buxue Tang (*Astragali radix* and *Angelicae sinensis* radix) for menopausal symptoms: A review	2017	10	#2
Lu et al. (2009)	Phthalide mono- and dimers from the radix of *Angelica sinensis*	2009	15	#3
Su et al. (2013)	Chemical fingerprinting and quantitative constituent analysis of Siwu decoction categorized formulae by uplc-qtof/ms/ms and hplc-dad	2013	7	#4
Liu E. H. et al. (2010)	Recent advances in quality control of traditional Chinese medicines	2010	21	#5
Liu et al. (2011)	Inhibitory effect of *n*-butylidenephthalide on neointimal hyperplasia in balloon injured rat carotid artery	2011	7	#7
Bunel et al. (2015)	Nephroprotective effects of ferulic acid, z-ligustilide and e-ligustilide isolated from *Angelica sinensis* against cisplatin toxicity *in vitro*	2015	6	#8
Zhou et al. (2015)	Beneficial effect of Danggui-Shaoyao-San, a traditional Chinese medicine, on drowsiness induced by chronic restraint stress	2015	4	#9

The remaining clusters are also worth mentioning, in particular, #5 “Quality control” and #8 “Ferulic acid.” The #5 “Quality control” cluster had 29 member articles with an average publication year of 2010. The most active citing paper (Liu E. H. et al., 2010) to this cluster was a review published in 2017 that summarized advances in TCM quality control. Quality control is extremely important to clarify the therapeutic effect of TCM and guarantee the clinical efficacy of TCM (Liu E. H. et al., 2010; Flower et al., 2014), and also is an essential research topic in the European Union’s Seventh Framework Programme (FP7) (Xu et al., 2013). The #8 “Ferulic acid” cluster also had 29 member articles with an average publication year of 2007. The most active citing paper (Bunel et al., 2015) was published in 2015 found that ferulic acid, which is one of the important compounds found in AS, exerts nephroprotective effects via decreasing cell death and improving cell regeneration. From above-mentioned analyses, it is clear that the knowledge base and research development of AS research has covered a variety of aspects including active compounds, biologic effects and mechanisms, prescription rule (DGBX), quality control, and analysis methods.

### Emerging Topics

**FIGURE 5:**
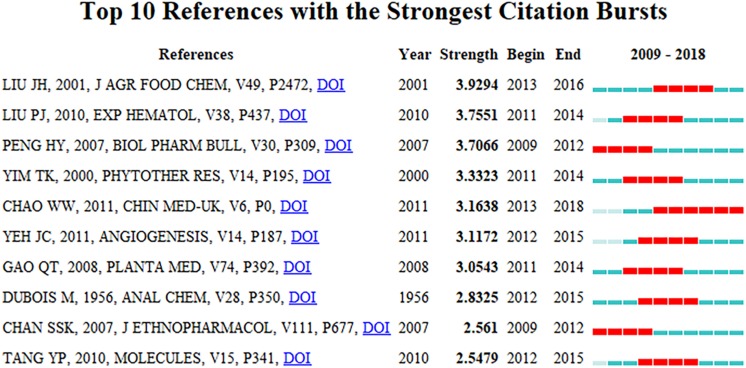
The top 10 *Angelica sinensis* articles with the strongest strength citation burst.

The top 10 references with the strongest strength citation burst identified by CiteSpace are presented in Figure 5. Papers with a high citation burst are paid much attention by peers in the scientific community, therefore emerging trends or topics within the AS research field were labeled in this study by AS publications with high citation bursts (Chen, 2017; Miao et al., 2018). Overall, the burst strength of the top 10 AS publications ranged from 2.5479 to 3.9294, while endurance strength ranged from 4 to 6 years, as indicated by the number of red squares. Among the top 10 papers, the citation burst of five publications (Dubois et al., 1956; Liu et al., 2001; Tang et al., 2010; Chao and Lin, 2011; Yeh et al., 2011) ended in 2015 or later, and therefore they reflect the most recent research topics in AS research and were selected for further discussion. The first paper as ranked by burst strength (Liu et al., 2001) was published in 2001 and the citation burst lasted for 4 years (2013–2016). The experiment analyzed the estrogenic activity of eight common plants for menopausal symptoms and found AS displays weak estrogen receptors binding, and the authors concluded that dietary supplements have potential for treating women with menopausal symptoms. Chao and Lin (2011) reviewed over 70 major compounds of AS and their biologic effects, and found that: (1) ferulic acid, butylidenephthalide and Z-ligustilide all have anti-inflammatory effects; (2) ferulic acid and polysaccharides have immunostimulatory effects; (3) butylidenephthalide has anti-cancer and anti-cardiovascular effects; and (4) Z-ligustilide has anti-cancer, neuroprotective and anti-hepatotoxic effects. The review was published in *Chinese Medicine* in 2011 and had the second strongest citation burst of the top five publications with the citation burst lasting for 6 years (2013–2018) from. The article with the third-highest citation burst (Yeh et al., 2011) was entitled “*The natural compound n-butylidenephthalide derived from the volatile oil of Radix Angelica sinensis inhibits angiogenesis in vitro and in vivo*,” was published in 2011, had a burst strength of 3.1172, and the burst lasted for 4 years (2012–2015). This article proved the effect of AS for inhibiting angiogenesis of *n*-butylidenephthalide via *in vitro* and *in vivo* experiments. In the paper with the fourth-highest citation burst (Dubois et al., 1956) the authors described an important method, phenol-sulfuric acid method, that is used for the analysis of various sugars and when combined with paper partition chromatography can be used to determine the chemical composition of polysaccharides and corresponding methyl derivatives. This paper had a burst strength of 2.8325, but, interestingly, it was published in 1956 and its burst lasted for 4 years from 2012 to 2015. One possible reason for this display in citation burst is that this method has been widely used to extract polysaccharides from AS in recent years. Finally, the article with the fifth-highest citation burst (Tang et al., 2010) was published in Molecules in 2010, with a burst strength of 2.5479 that lasted from 2012 to 2015. This study analyzed and compared bioactive phthalides in essential oils from AS, CX, SW, and Fo-Shou-San, and concluded that comparative analyses of compounds and biologics are helpful to elucidate active components and prescription mechanisms of TCM. The result of the emerging topics analysis reveals that exploring active components of AS and their pharmacological effects are the current major topics in the AS research field.

### Strengths and Limitations

Our research has several strengths to note. First, this paper is the first use of scientometric method to systematically analyze AS studies, which can provide references for researchers in the AS field. Second, two widely used scientometric software tools were employed to conduct the present study at the same time, both tools were designed by well-known information scientists (Garfield et al., 2006; Chen, 2017). However, similar to other scientometric studies, there are several limitations are to note in our study. First, we only searched the WoS database, and did not searched other large medical databases such as PubMed, Scopus, or Embase. However, it should be noted that WoS is the most commonly used database for scientometric studies (Miao et al., 2018; Chen et al., 2019; Lu et al., 2019). Second, all data were extracted by software tools, unlike systematic review or overview where data were extracted by two or more reviewers manually (Li et al., 2015; Pan et al., 2018; Cai et al., 2019). Therefore, data used to support our results may have bias. Third, we did not analyze core authors, who publish the most articles related to AS due to the possibility of homonyms, but that information would be helpful so that researchers could develop cooperative relationships with those productive institutions. Finally, we did not include papers published in 2019 on AS, as this data was incomplete at the time of our database search.

## Conclusion

We employed CiteSpace and HistCite to identify intellectual base and emerging trends (active components of AS and pharmacological actions) on AS publications. Most articles in this field were published in China, with Nanjing University of Chinese Medicine contributing the most publications. The core journal in AS research was the *Journal of Ethnopharmacology*. A holistic view of the development of AS research over the past decade was provided using quantitative and qualitive methods, which can provide guidance for scientists in AS research field.

## Data Availability Statement

All data generated for this study are included in the article.

## Author Contributions

CL, MiL, and KY designed this study. CL and MiL ran the search strategy. WS and YY collected data, MeL and XD re-checked data. CL and MiL performed analysis, WS and HL re-checked. CL wrote the manuscript. All listed authors reviewed and revised the manuscript.

## Conflict of Interest

The authors declare that the research was conducted in the absence of any commercial or financial relationships that could be construed as a potential conflict of interest.

## Footnotes

1 https://jcr.clarivate.com/JCRJournalHomeAction.action

2 http://www.nhc.gov.cn/guihuaxxs/s10748/201905/9b8d52727cf346049de8acce25ffcbd0.shtml
